# Impact of IYCF practices, as measured by national nutrition surveys from 2018 to 2022, on stunting and under-5 survival in Burkina Faso: a LiST analysis

**DOI:** 10.3389/fnut.2024.1504564

**Published:** 2025-01-08

**Authors:** Natacha Kere, Ella W. R. Compaore, Youssouf Keita, Daniel S. Ouedraogo, Souleymane Tirogo, Estelle A. Bambara, Z. Thierry Coulibaly, Mamoudou H. Dicko

**Affiliations:** ^1^Laboratory of Biochemistry, Biotechnology, Food Technology and Nutrition (LABIOTAN), Department of Biochemistry-Microbiology, Joseph KI-ZERBO University, Ouagadougou, Burkina Faso; ^2^Secrétariat Technique chargé de la multisectorialité pour la nutrition (ST-NUT), Ministère de la Santé, Ouagadougou, Burkina Faso; ^3^Global Finance Facility, World Bank, Bamako, Mali; ^4^Direction d ela nutrition (DN), Ministère de la Santé, Ouagadougou, Burkina Faso; ^5^Plateforme Nationale d’Information pour la Nutrition (PNIN), Ouagadougou, Burkina Faso

**Keywords:** children 0–5 years old, chronic malnutrition, impact, infant, IYCE, LiST, nutritional survey, stunting

## Abstract

**Introduction:**

Burkina Faso is facing a serious public health problem of chronic malnutrition and mortality in children under the age of 5. To tackle this situation, a number of child nutrition interventions have been implemented. This study aims to assess the impact of these interventions on the nutritional status of children aged 0–5 years between 2018 and 2022.

**Methods:**

This study is a modeling of the impact of changes in the coverage of interventions with known effect sizes, using the LiST (Lives Saved Tool). The interventions used concern infant and young child feeding, the coverage of which was measured by the 2018 to 2022 editions of the NNS conducted in Burkina Faso using the SMART methodology. It covered the national level, the Center region and the Sahel region. Extracted intervention coverage levels were entered into LiST, according to six projections in two scenarios (scenario 1 and 2). The modeling results visualized in LiST were exported to Excel for formatting into tables and/or graphs.

**Results:**

The measured changes in the levels of infant and young child feeding interventions included in this study led to a reduction in the number of cases of chronic malnutrition in children under 6 months of age in the second scenario projections. However, no cases of chronic malnutrition were prevented among children in the 6–59 month age group under any of the six projections. On the contrary, opportunities to save cases of chronic malnutrition have been missed. These missed opportunities amount to 64,880 in scenario 1 and 39,556 in scenario 2 at national level. Nevertheless, 920 lives were saved nationwide, 232 in the Center region and 202 in the Sahel region.

**Conclusion:**

The study highlighted the need to strengthen the implementation of IYCF interventions, coupled with a significant improvement in the quality of NNS data collection, given the up and down nature of the indicators, which makes them difficult to use for decision-making.

## 1 Introduction

Malnutrition in children under the age of five is a global public health problem that can contribute to the failure to achieve sustainable development goals (1) particularly in developing countries. Stunting, as well as other forms of childhood undernutrition, remains a public health problem. In fact, 148.1 million children under the age of 5 were stunted worldwide in 2022. Most of these children live in Asia (52%) and Africa (43%) (2).

Burkina Faso, like other African countries, remains one of the countries most affected by chronic malnutrition and under-five mortality. Although the trend is downwards, the prevalence of chronic malnutrition remains high. In 2021, it was 21.6% (3), which is still high compared to the prevalence in the West African region, which was 30% (4).

Malnutrition has many short- and long-term consequences. An estimated 45% of deaths in children under 5 are linked to malnutrition (5). Every year, it contributes to the deaths of 5.6 million children worldwide (6). Furthermore, stunting, a form of malnutrition, is a huge public health burden with long-term consequences for the health of children in low- and middle-income countries (7). It can lead to delayed cognitive development and increased morbidity and mortality (8). In addition, stunting and other forms of undernutrition, when they occur early in life, can predispose children to overweight and non-communicable diseases later in life (9).

According to the conceptual framework of malnutrition, it is caused not only by disease, but also by inadequate food rations (10). Moreover, the incidence of stunting generally peaks around 6 to 23 months of age, due to the transition from exclusive breastfeeding to the introduction of complementary foods that may be of poor nutritional quality (11, 12).

To effectively tackle the problem of malnutrition, several strategic interventions are implemented. However, identifying and prioritizing interventions to reduce the burden of morbidity and mortality among women and children is a crucial task for governments and ministries of health (13). Among the interventions implemented in the fight against malnutrition are infant and young child feeding (IYCF) interventions. Evidence shows that interventions such as breastfeeding and complementary feeding can reduce child mortality by 13% (14). Based on scientific evidence and international recommendations, Burkina Faso has drawn up a national plan for scaling up IYCF practices to 2013–2025 (15). Since 2014, infant and young child feeding interventions have been implemented in the different regions of the country even though area coverage is not the same in all regions.

The Government of Burkina Faso is committed to strengthening nutrition interventions. Since 2009, this commitment has been reflected in the implementation of an effective monitoring mechanism, supported by the annual conduct of the National Nutrition Survey (NNS) using the SMART (Standardized Monitoring and Assessment of Relief and Transition) methodology. The NNS aims to provide not only current data on the nutritional situation, but also on IYCF practices. Indicators of IYCF practices measured in the NNS include early initiation of breastfeeding, exclusive breastfeeding, continued breastfeeding for up to 1 year, continued breastfeeding for up to 2 years, minimum dietary diversity, minimum meal frequency and minimum acceptable diet. Thanks to national nutritional surveys, the prevalence of different forms of malnutrition and the level of infant and young child feeding indicators are known for each region of the country.

Despite the regular collection of data on the coverage of IYCF interventions with known effect sizes, knowledge of the impact of measured changes in children under five remains largely unknown. Hence the interest of this study, which aims to assess the modeled impact of changes in the level of IYCF indicators between 2018 and 2022 measured by the NNS on stunting and child survival through the LiST tool, a mathematical model that uses the best available data on population, cause of death, intervention, effectiveness and coverage to estimate the impact of change in intervention coverage on malnutrition and under-five and maternal mortality (16). Specifically, the aim was to assess the impact of IYCF interventions as measured on (i) the reduction in the number of cases of chronic malnutrition among children under five; (ii) the number of lives saved among children under five; and (iii) the reduction in the mortality rate among children under five.

## 2 Methodology

### 2.1 Study outline

This study is a retrospective evaluation of the impact of IYCF interventions measured by NNS between 2018 and 2022 on chronic malnutrition and under-five mortality in Burkina Faso using the LiST tool. LiST is a mathematical model, part of the Spectrum software modules, which uses the best available data on population, causes of death, interventions, effectiveness and intervention coverage to model the impact of changes in intervention coverage on mortality (maternal, neonatal and infant), malnutrition (acute and chronic) and low birth weight (17). It serves as an essential tool for evaluation, strategic planning and advocacy to strengthen informed decision-making and the implementation of effective strategies. Evaluation is defined as retrospective analyses that determine changes in outcomes over time, including assessments of government-led national plans to reduce mortality, as well as evaluations of smaller-scale health programs (17).

The study took place between December 2023 and May 2024.

### 2.2 Study framework

This study covered two levels of analysis: national and regional.

For the modeling, data were extracted from NNS reports from 2018 to 2022. These reports include data for the national level, as well as for each of the country’s 13 regions. So, for this study, in addition to the national level, two regions were modeled. These regions were chosen on the basis of the prevalence of chronic malnutrition recorded between 2018 and 2022. The region with the highest and lowest prevalence of chronic malnutrition was selected. Of the country’s 13 health regions, the Sahel and Center regions were selected. The target population was all children aged 0–59 months in Burkina Faso.

### 2.3 Data used

For the modeling, data extracted from NNS reports from 2018 to 2022 were used. These are the prevalences of acute and chronic malnutrition, as well as the level of five IYCF indicators: early initiation of breastfeeding, exclusive breastfeeding, continued breastfeeding for up to 1 year, continued breastfeeding for up to 2 years, and the introduction of complementary foods. In addition, standard national infant mortality rates as published by the United Nations Inter-Agency Group for Child Mortality Estimation (IGME) were used (18).

All other default values in LiST were kept unchanged, as were the levels of other interventions not modeled in this study. The structure of causes of death, the effectiveness of interventions and the socio-demographic characteristics of the population of Burkina Faso and the regions selected for modeling were also maintained.

### 2.4 Modeling and visualization in LiST

LiST version 5.621 was used for data modeling.

LiST is a mathematical model that calculates the impact of a change in intervention coverage on intervention-sensitive causes of mortality using the following formula:

$\begin{array}{l} \mathrm{Impact}=\mathrm{Change}\;\mathrm{in}\;\mathrm{intervention}\;\mathrm{coverag}e^{*}\mathrm{Effectivenes}s^{*}\\ \mathrm{Fraction}\;\mathrm{affected}\text{.} \end{array}$

Impact is calculated by reassessing the input sensitive to an intervention or package of interventions following the scaling up of the intervention or package of interventions between the base year and the target year. In this study, the impact was calculated between 2018 and 2022.

The initial aim of LiST is to estimate the impact of scaling up interventions on under-five mortality (14). By impact, we mean the number of lives saved, the reduction in mortality rates (neonatal, infant/juvenile, maternal) and the reduction in malnutrition rates (chronic, acute). The number of lives saved in this tool corresponds to the number of deaths avoided.

In order to understand the impact of IYCF interventions as measured by NNS on under-five mortality through LiST, it was necessary to create the following scenarios. Two types of scenario were therefore devised: scenario 1 and scenario 2. In the first scenario, data extracted from NNS reports were entered as reported, without modification or adjustment. In the second, so-called optimistic scenario, only positive changes recorded by NNS were taken into account, with decreases excluded.

In the first scenario, the first projection includes data from the national level (National 1). The second projection is a scenario in which data from the region with the highest prevalence of chronic malnutrition, the Sahel, have been captured (Sahel 1). As for the third projection, it contains data from the Center region, the region with the lowest prevalence of chronic malnutrition (Center 1). National 1, Center 1 and Sahel 1 are the projections of the first scenario. The extracted data are shown in the Table 1.

**Table 1:** Levels of interventions coverage measured by national nutrition surveys from 2018 to 2022 at national and regional levels.

Indicators	Year	Center	Sahel	National
Early breastfeeding	2018	59.4	64.6	59.5
	2019	57.4	70.4	59.1
	2020	49.6	27	63.4
	2021	52.4	–	62.1
	2022	69.5	–	–
Exclusive breastfeeding	2018	30.9	48.7	55.8
	2019	34.5	67.9	59
	2020	45.8	61.1	64.3
	2021	64.1	–	69.6
	2022	72,5	–	–
Continued breastfeeding at age 1	2018	79.5	95.7	92.4
	2019	96.5	98	98.1
	2020	–	89.4	96.4
	2021	100	–	95.6
	2022	89.6	–	–
Continued breastfeeding at age 2	2018	–		
	2019	–	77.3	80.5
	2020	–	–	80.1
	2021	–	–	72.4
	2022	–	–	–
Timely introduction of food supplements	2018	–	67.8	70.8
	2019	–	69.7	61.6
	2020	–	–	77.4
	2021	31.8	–	63
	2022	–	–	–

The unavailability of data for certain years of the analysis period and the oscillation in the level of certain IYCF indicators included in the study from 1 year to the next led to the prior processing of data extracted from NNS reports.

The second scenario was composed of National 2, Center 2 and Sahel 2.

The Table 2 summarizes the processing carried out according to the scenarii to compensate for the unavailability of data in a given year.

**Table 2:** Description of scenarios’ construction by intervention included and by level.

Indicators	Data processing
Early breastfeeding	**National 1**: Data extracted from the NNS reports were used from 2018 to 2021. For 2022, due to lack of data, the 2021 value has been used. **Center 1**: These are the values extracted from the NNS reports that have been entered from 2018 to 2022. **Sahel 1**: Data extracted from NNS reports were captured from 2018 to 2020. The 2020 value was rolled over into 2021 and 2022 to fill the data gap for these 2 years. **National 2**: An interpolation was carried out between 2018 and 2021 and the resulting step size was 1.3. Using this step, the value for 2022 was calculated and the value obtained was 64.7. **Center 2**: An interpolation was made between 2018 and 2022 and the resulting step size was 2.5. **Sahel 2**: An interpolation was made between 2018 and 2020. The step size obtained was 2.9. Using this step, the values for 2021 and 2022 were calculated.
Exclusive breastfeeding for the first 6 months	**National 2**: An interpolation was made between 2018 and 2021 and the step obtained was 4.6. Using this step, the value for 2022 was calculated. As the rates for the different types of breastfeeding were greater than 100, a calculation was made to obtain 100 as the sum of all types of breastfeeding. **Center 2**: An interpolation was made between 2018 and 2022 and the same procedure was followed as for National 2. S**ahel 2**: An interpolation was made between 2018 and 2020. The step size obtained was used to calculate the value for 2021 and 2022.
Continued breastfeeding at age 1	**National 2**: An interpolation was made between 2018 and 2021. The step size obtained was 1.9. With this step, the value for 2022 was calculated. Center 2: An interpolation was made between 2018 and 2022. The step size obtained was 5.1. **Sahel 2**: An interpolation was made between 2018 and 2020. A step size of 1.1 was obtained. With this step, the values for 2021 and 2022 were deduced.
Continued breastfeeding at age 2	**National 2**: In 2018, the default value was used, as data was not collected for this year. Subsequently, an interpolation was made between 2018 and 2021, and the value found was 90.5 for all years. As a result, this value was repeated in 2022. **Center 2**: This is the default value (90.5) which is maintained for all years, as the indicator was not collected from 2018 to 2022. **Sahel 2**: The default value (91.9) has been maintained for all years, as there was only one data point in the 2019 NNS report.
Timely introduction of food supplements	**National 2**: Interpolation was performed between 2018 and 2021 and the value for 2022 was deduced based on the step obtained. **Center 2**: The default value (5.2) is maintained, as there is only one data point for 2018, which is 31.8. **Sahel 2**: Existence of only two data points for 2018 and 2019. No interpolation has been done as we only have 2 data points. To obtain data values for subsequent years, the step was calculated between 2018 and 2019. With this step, the values for 2020, 2021 and 2022 were calculated and were 71.6, 73.5 and 75.4, respectively.

For each projection, 2018 was used as the base year and 2022 as the target year.

To estimate the impact of IYCF interventions on the prevalence of chronic malnutrition, the “direct entry” options for chronic and acute malnutrition were unchecked at the time of configuration.

An analysis of the evolution of each IYCF indicator was carried out from 2018 to 2022, calculating the average annual rate of reduction (AARR) and the average annual growth rate (AAGR) to assess the overall direction of evolution.

The results visualized in LiST were exported to Excel for formatting in the form of tables and/or graphs. Charts and tables were also used to better represent the results modeled by LiST.

## 3 Results

### 3.1 Data processing results

Data extracted from NNS reports from 2018 to 2022 for analysis were pre-processed to cope with data unavailability and data oscillation from year to year. The data obtained and entered into LiST are presented in the Table 3.

**Table 3:** Interventions coverage levels by projection and scenario at national and regional levels.

Indicators	Année	Projections: Scenario 1			Projections: Scenario 2		
		Center 1	Sahel 1	National 1	Center 2	Sahel 2	National 2
Early breastfeeding	2018	59.4	64.6	59.5	59.4	64.6	59.5
	2019	57.4	70.4	59.1	61.9	67.5	60.8
	2020	49.6	27	63.4	64.4	70.4	62.1
	2021	52.4	27	62.1	66.9	73.3	63.4
	2022	69.5	27	62.1	69.5	76.2	64.7
Exclusive breastfeeding for the first 6 months	2018	30.9	48.7	55.8	30.9	48.7	55.8
	2019	34.5	67.9	59	41.3	58.3	60.4
	2020	45.8	61.1	64.3	51.7	67.9	65
	2021	64.1	61.1	69.6	62.1	77.5	69.6
	2022	72.5	61.1	69.6	72.5	87.1	72.4
Continued breastfeeding at age 1	2018	79.5	95.7	92.4	79.5	95.7	92.4
	2019	96.5	98	98.1	84.6	96.8	94.3
	2020	96.5	89.4	96.4	897	98	96.2
	2021	100	89.4	95.6	94.8	99.1	98.1
	2022	89.6	89.4	95.6	100	100	100
Continued breastfeeding at age 2	2018	90.5	91.9	90.5	90.5	91.9	90.5
	2019	90.5	77.3	80.5	90.5	91.9	90.5
	2020	90.5	77.3	80.1	90.5	91.9	90.5
	2021	90.5	77.3	72.4	90.5	91.9	90.5
	2022	90.5	77.3	72.4	90.5	91.9	90.5
Timely introduction of food supplements	2018	5.2	67.8	70.8	5.2	67.8	70.8
	2019	5.2	69.7	61.6	5.2	69.7	73
	2020	5.2	69.7	77.4	5.2	71.6	75.2
	2021	31.8	69.7	63	5.2	73.5	77.4
	2022	31.8	69.7	63	5.2	75.4	79.6

### 3.2 Impact of IYCF interventions on under-nutrition in children under 5 years of age

#### 3.2.1 Number of cases of chronic malnutrition prevented

**Figure 1:**
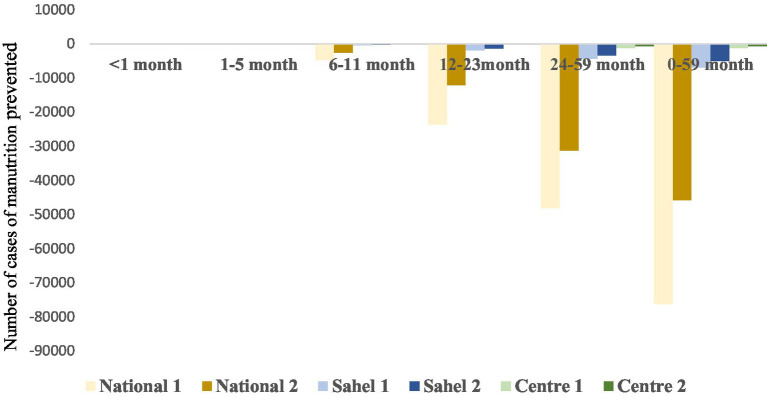
Number of cases of chronic malnutrition prevented in children aged 0–59 months.

The LiST analysis shows that cases of chronic malnutrition have been avoided among children aged under 1 month and between 1 and 5 months in all second scenario projections (National 2, Center 2 and Sahel 2) and also for National 1 and Center 1. However, the results show that no cases of chronic malnutrition were averted among children in the 6–11 months, 12–23 months, 24–59 months age groups, and overall among children aged 0–59 months, for all six projections and whatever the scenario. Indeed, up to 76,289 and 45,768 opportunities were missed overall among children aged 0–59 months for National 1 and National 2, respectively (Figure 1).

#### 3.2.2 Prevalence of chronic malnutrition

**Figure 2:**
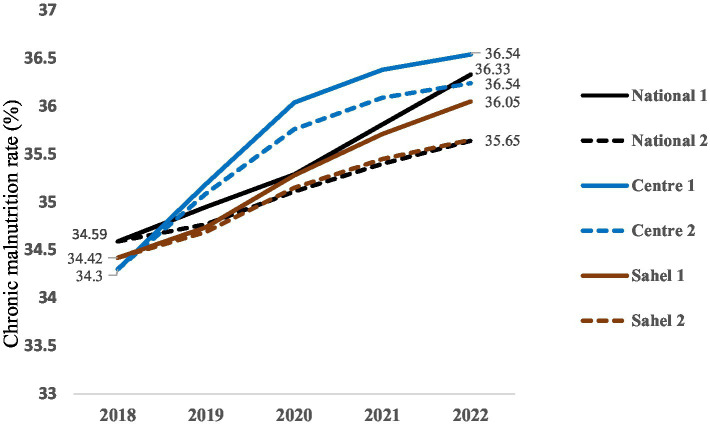
Prevalence of chronic malnutrition.

The results show that the modeled interventions did not lead to a reduction in chronic malnutrition prevalences between 2018 and 2022 for the different projections. At national level, a large variation was observed between the prevalence of chronic malnutrition obtained for National 1 and that obtained for National 2. However, the prevalences obtained with the different scenarii are similar for the Center and Sahel regions (Figure 2). Furthermore, according to the results, prevalences of chronic malnutrition increase less rapidly under the optimistic scenario that only takes positive changes into account.

#### 3.2.3 Number of lives saved

LiST allows us to calculate the number of lives saved, which corresponds to the number of deaths avoided per intervention.

The results of this study show that the lives of children under the age of five have been saved, even though most of these children are suffering from chronic malnutrition. In fact, for all the second scenario projections (National 2, Center 2 and Sahel 2) and for Center 1, lives were saved. For the Center region, 232 lives were saved for the first scenario and 219 for the second scenario from 2018 to 2022.

**Figure 3:**
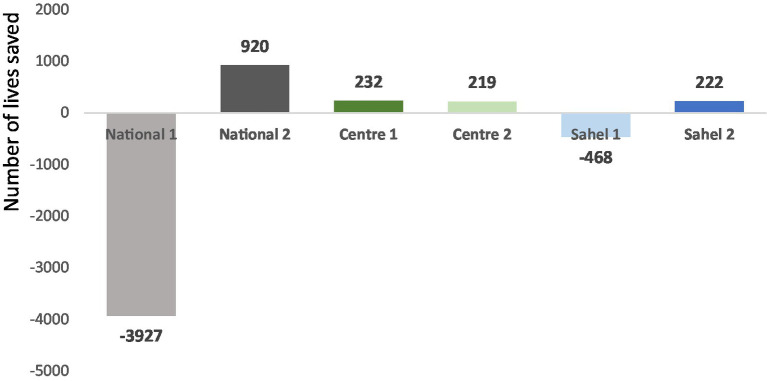
Number of lives saved.

Although many lives were saved between 2018 and 2022, some crucial opportunities were missed, particularly in the first scenario projections. In other words, in these projections, deaths of children under five could not be avoided, as evidenced by the negative values. For the National 1 and Sahel 1 projections, more than 3,900 and 468 deaths, respectively, could not be avoided (Figure 3).

#### 3.2.4 Mortality rate

**Figure 4:**
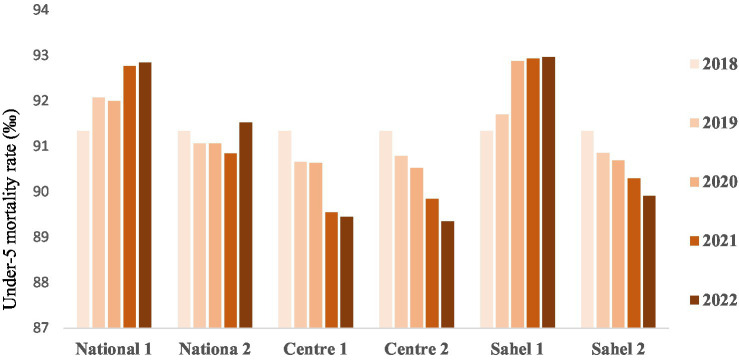
Under-five mortality rate (‰ Live births).

The results show a decrease in the mortality rate from 2018 to 2022 for the central region regardless of the type of scenario, as well as for the second scenario for the Sahel region. However, the results show an increase in the mortality rate for the first scenarii at national level and for the Sahel region (Figure 4).

## 4 Discussion

The present study was based on modeling with the LiST tool and aims to assess the impact of IYCF interventions measured by NNS on reducing the number of cases of chronic malnutrition, the number of lives saved and reducing the mortality rate among children under five. Analysis with LiST also makes it possible to identify missed opportunities and prioritize interventions according to their impact on maternal, newborn and child health (19).

Overall, this modeling shows that projections based on positive changes in indicator levels do better than taking into account all estimates as measured by NNS. Furthermore, this analysis showed a limited impact of the IYCF interventions implemented to address the issue of child malnutrition.

### 4.1 Impact on reducing chronic malnutrition

The results show that cases of chronic malnutrition were prevented in children under 6 months of age by all the projections in the second scenario (National 2, Center 2 and Sahel 2) and also for National 1 and Center 1. These results could be explained by the good practice of exclusive breastfeeding among these children. Indeed, according to UNICEF’s causal diagram of malnutrition, inadequate feeding practices are a cause of malnutrition (20). To address this issue, the World Health Organization recommends that infants should be exclusively breastfed for the first 6 months of life. Another explanation would be the effect of breastfeeding on the incidence of diarrhea, which in turn has an impact on the appearance of stunted growth and on mortality (5). However, cases of chronic malnutrition among infants aged 0–5 months in Sahel 1 could not be prevented. The security and humanitarian crisis in this part of the country, resulting in massive population displacements and reduced access to health and nutrition services, may play a role in this outcome.

Inappropriate IYCF practices, such as late introduction of complementary foods, poor dietary diversification and failure to respect the conditions for adequate complementary feeding, expose children to malnutrition. Indeed, the absence of exclusive breastfeeding for the first 6 months, followed by a lack of varied and nutritious complementary foods, can lead to stunted growth, emaciation and micronutrient deficiencies (21, 22). The nutritional needs of children aged 6 to 23 months are greater than at any other time in their lives, making them particularly vulnerable to nutritional deficiencies and growth disorders (23).

In addition, inaccurate measurement of indicator levels during the NNS would explain these results. In addition, imprecise measurement of indicator levels during the NNS would explain these results. During the NNS, the level of IYCF indicators is obtained by interviewing mothers/caretakers of children aged 6 to 23 months. The 24-h recall method is used for this purpose. If the questions are not asked as described in the questionnaires to the mothers, this could have repercussions on the mothers’ answers and, in turn, on the level of the indicators.

### 4.2 Number of lives saved

The results show that lives were saved for all the projections in the second scenario (National 2, Center 2 and Sahel 2) and for Center 1, despite cases of chronic malnutrition in certain age groups. This could be due to the adoption by some mothers of recommended feeding practices for infants and young children, such as the practice of exclusive breastfeeding, the timely introduction of complementary foods, and the continuation of breastfeeding beyond 6 months.

Despite the lives saved during the period of our study as a result of the five interventions, opportunities were missed, especially for the projections in the first scenario, even though the IYCF interventions were implemented. These results do not corroborate those found by a Lancet study which showed that around 2.4 million children each year could be saved by a package of effective nutritional interventions including breastfeeding, complementary feeding, vitamin A and zinc supplementation (14). The missed opportunities observed could be explained by the fact that the average change in indicator levels was downward over the analysis period, rather than increasing significantly from 1 year to the next, as evidenced by the AARR and AAGR, which showed negative slopes. In addition, these results could be explained by the small sample size used to calculate IYCF indicators, and by the low target and geographical coverage of IYCF interventions in the country. In fact, data collection for the calculation of IYCF indicators is integrated with anthropometric data collection during NNS, and the sample size calculation takes into account the target for anthropometric data collection, which is 0 to 59 months, and not the target for the calculation of IYCF indicators, which is 0 to 23 months. Furthermore, the sample size used to calculate IYCF indicators by region is small compared to the number of children aged 0 to 23 months in the region, which could explain why lives were not saved. In addition, not all regions are covered by the two implementation components (health training and community level) of IYCF interventions, as envisaged in the IYCF practice scaling plan (15). IYCF learning and follow-up groups, which are mother-to-mother groups, have not been set up in all health zones, yet they provide a platform for pregnant women and mothers of children aged 0 to 23 months to adopt the advice given at both health facility and community levels.

### 4.3 Mortality rates

Although the interventions considered in our study are not the only ones that save the lives of children under the age of five, an increase in the mortality rate was noted for the national level and the Sahel. This could be explained by an increase in the rate of chronic malnutrition or inappropriate feeding practices. The low geographical and target coverage of the IYCF interventions implemented could also explain these results. Indeed, the results of a study on infant and child feeding indicated that inappropriate feeding practices can have profound consequences on the growth, development and survival of infants and children, particularly in developing countries (24).

The results of this study are not in line with some evidence that IYCF interventions prevent deaths in target children. Also, a review of evidence-based interventions for improving maternal and child nutrition showed that the interventions with the greatest potential impact on under-five mortality are management of severe acute malnutrition, preventive zinc supplementation and promotion of breastfeeding (5).

### 4.4 Study limitations

Despite the relevance of the subject, this analysis has some significant limitations, such as the unavailability of data. Indeed, due to the humanitarian and security crisis in the country, data on certain indicators were not available. In addition, only a very limited number of interventions were included in the study, out of a large number with a positive impact on chronic malnutrition and child survival.

## 5 Conclusion

The study first highlighted a very significant variation in the level of indicators of IYCF practices measured by NNS between 2018 and 2022 at national level and in the two regions. Secondly, overall, projections based on positive variations in indicator levels do better than taking into account the data as provided by NNS. Indeed, lives were saved for all second scenario projections (National 2, Center 2 and Sahel 2) and for Center 1. Although lives were saved, at national level, missed opportunities were noted and amounted to over 3,900 in scenario 1 and over 460 for the scenario including the best positive changes recorded by NNS. In addition, a decrease in the mortality rate was observed for the Center region, and an increase for the national level and for the Sahel region.

In view of the up-and-down evolution of IYCF indicators according to NNS reports, making it difficult to assess their impact, it is necessary to strengthen the implementation of IYCF interventions at both health facility and community level. Particular attention must therefore be paid to data collection on IYCF interventions, and if possible, specific surveys must be carried out on these interventions.

## Data Availability

The original contributions presented in the study are included in the article/supplementary material, further inquiries can be directed to the corresponding author.
